# Supplementary material for: The bearing capacity of asteroid (65803) Didymos estimated from boulder tracks
Source: Nat Commun. 2024 Jul 30;15:6204. doi: 10.1038/s41467-024-50149-8 (PMC11289458; doi:10.1038/s41467-024-50149-8)
Supplement: Supplementary file 1 — Supplementary Information [file 41467_2024_50149_MOESM1_ESM.pdf]

# - Supplementary Information -

The bearing capacity of asteroid (65803) Didymos estimated from boulder tracks

J. Bigot[1], P. Lombardo[1], N. Murdoch[1*], D. J. Scheeres[2], D. Vivet[1], Y. Zhang[3], J. Sunshine[4], J. B. Vincent[5], O. S. Barnouin[6], C. M. Ernst[6], R. T. Daly[6], C. Sunday[4], P. Michel[7], A. Campo-Bagatin[8], A. Lucchetti[9], M. Pajola[9], A. S. Rivkin[6] and N. L. Chabot[6]

*Corresponding author. Email: naomi.murdoch@isae.fr

[1] Institut Supérieur de l'Aéronautique et de l'Espace (ISAE-SUPAERO), Université de Toulouse, Toulouse, France

[2] University Colorado, Boulder, USA

[3] Climate & Space Sciences and Engineering, University of Michigan, Ann Arbor, MI, USA

[4] University of Maryland, USA

[5] DLR, Germany

[6] Johns Hopkins Applied Physics Laboratory, USA

[7] Université Côte d'Azur, Observatoire de la Côte d'Azur, CNRS, Laboratoire Lagrange, France

[8] University of Alicante, Spain

[9] INAF-OAPD Astronomical Observatory of Padova, Italy

# - Supplementary Figures -

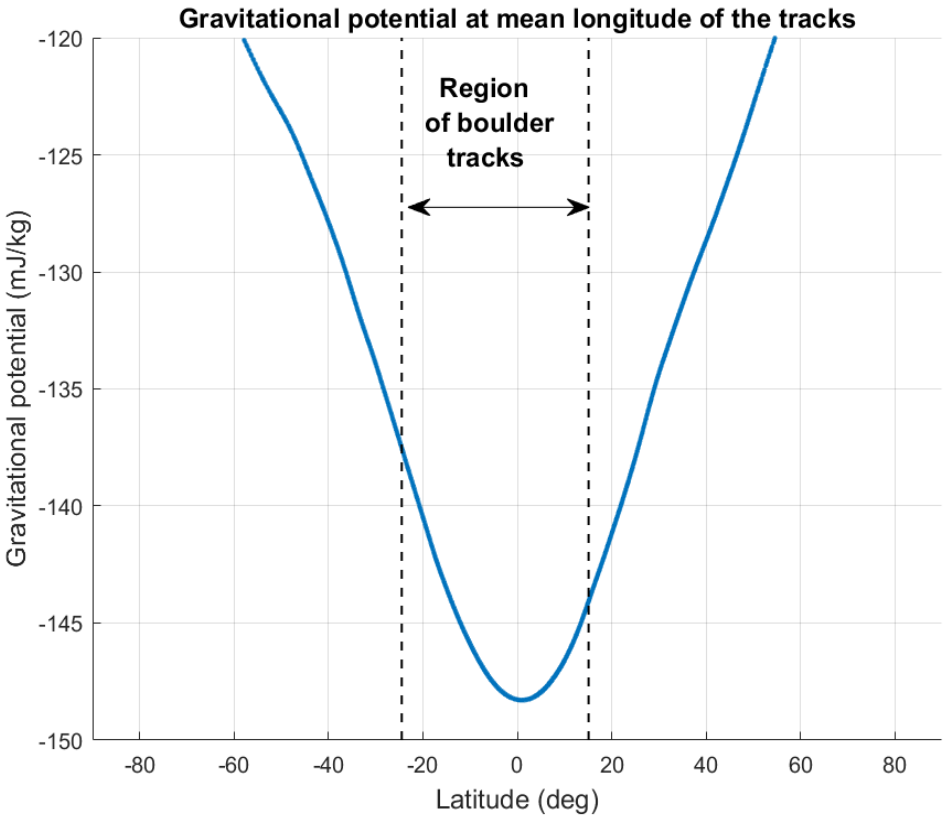

Supplementary Figure 1. Gravitational potential versus latitude at mean longitude of the boulder tracks. The region of boulder tracks is defined by the maximum latitudes found for the tracks.

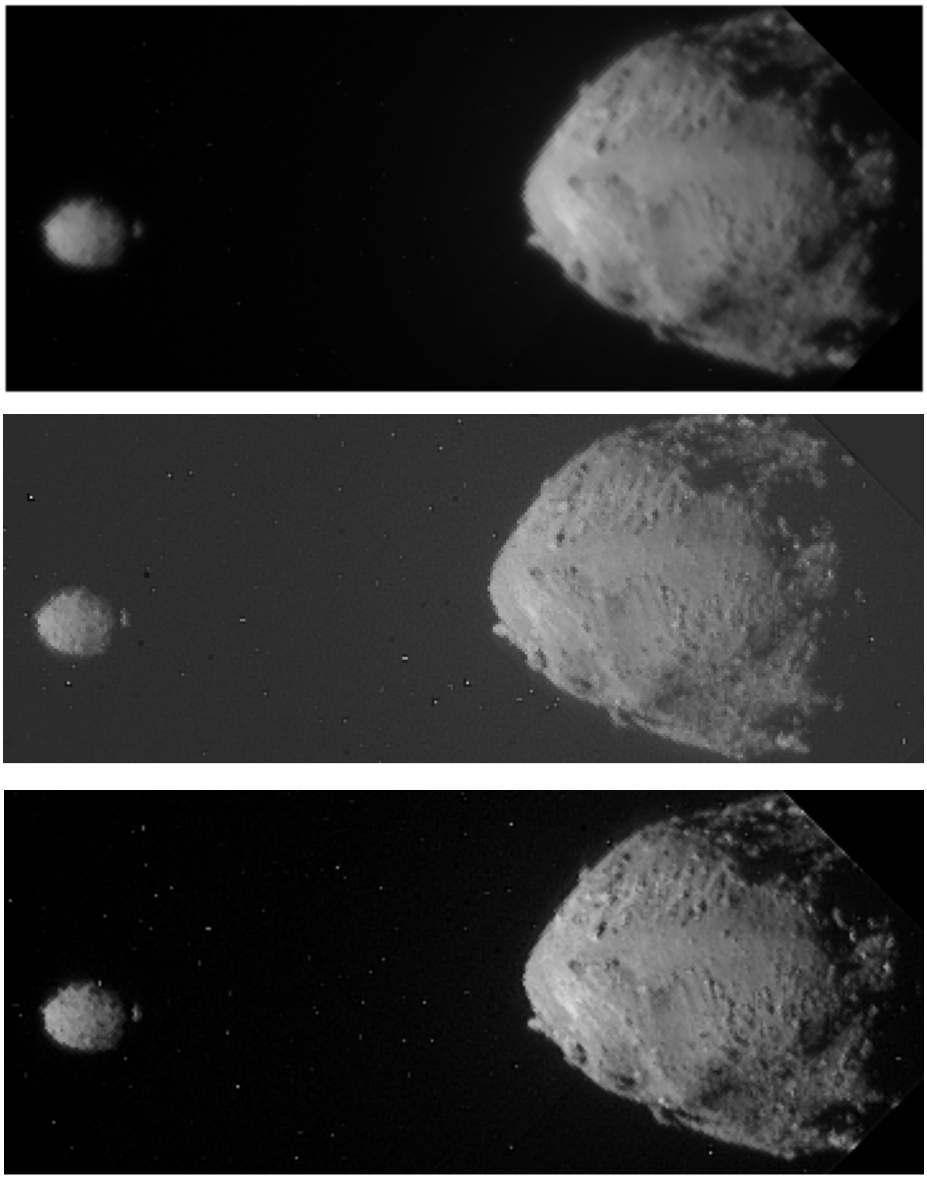

Supplementary Figure 2. Influence of image filtering. (Top) An example raw image, (Middle) High Boost filtered image and (Bottom) Laplacian filtered image. The image used here is DRACO image 22206 (pixel scale: 4.43 m).

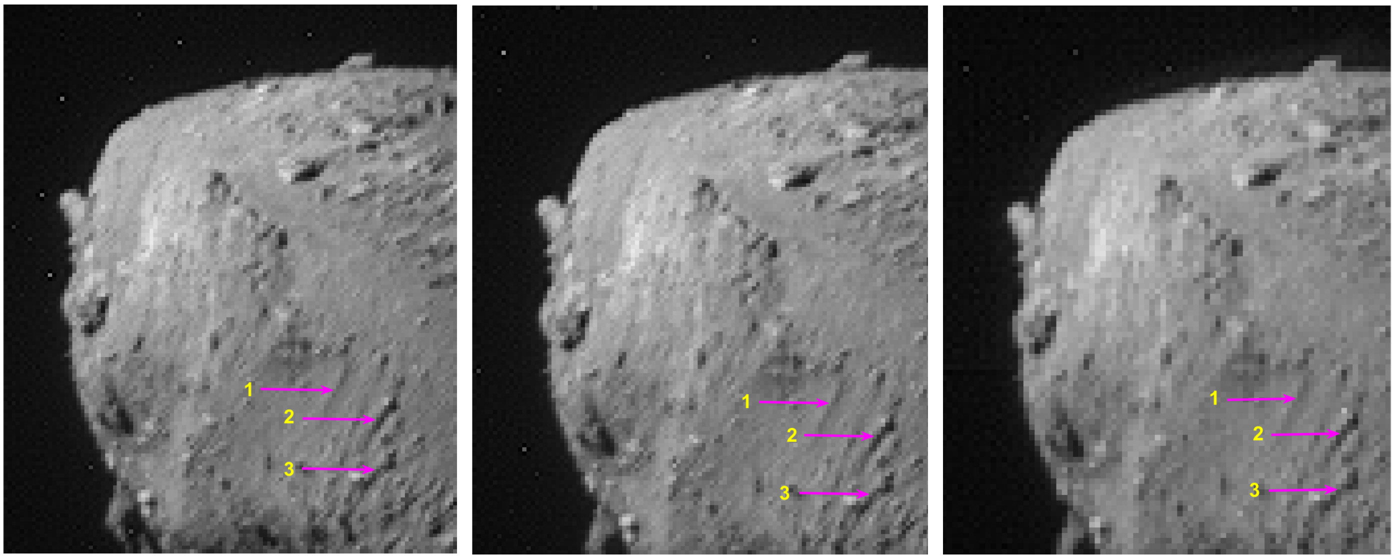

(a) (b) (c)

Supplementary Figure 3. The locations of tracks 1, 2, and 3 are indicated with the purple arrows in three different DRACO images, each with a different resolution: (a) image 10933 (pixel scale: 3.47 m), (b) image 05363 (pixel scale: 3.38 m) and (c) image 22206 (pixel scale: 4.43 m).

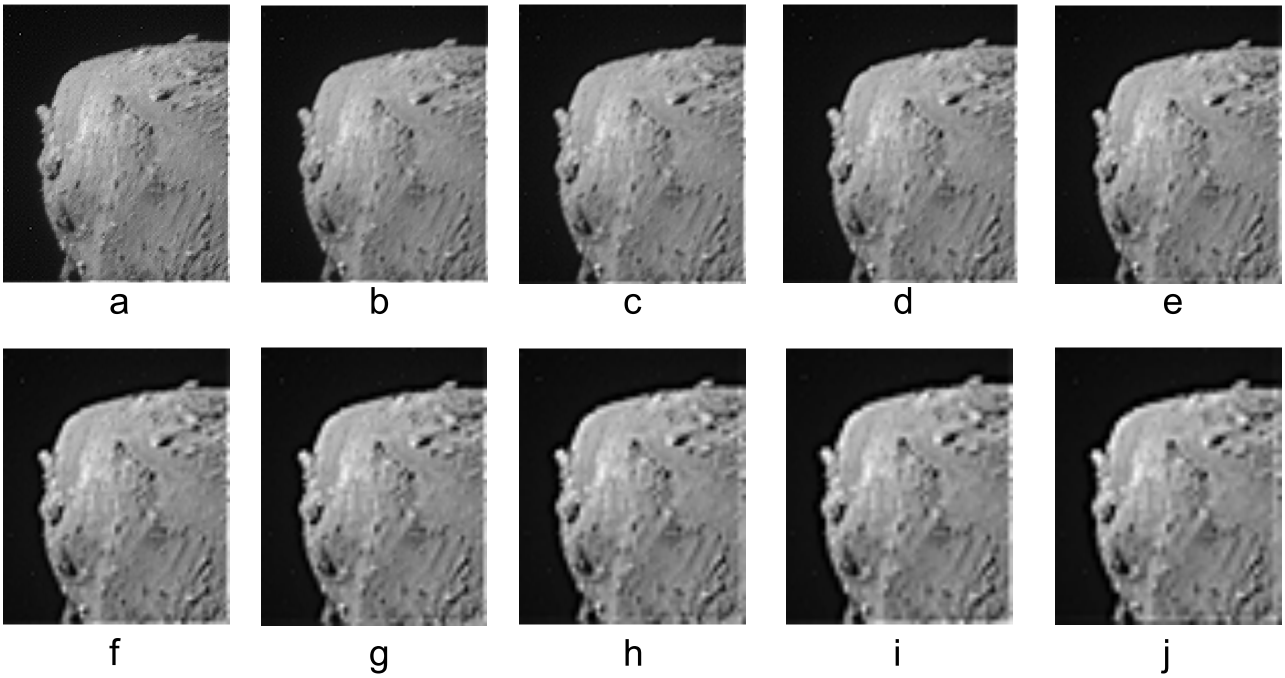

Supplementary Figure 4. (a) The original image 05363 with a pixel scale of 3.38 m. Degraded versions of image 05363 with different pixel scale of (b) 3.88 m, (c) 4.38 m, (d) 4.88 m, (e) 5.38 m, (f) 5.88 m, (g) 6.38 m, (h) 6.88 m, (i) 7.38 m, and (j) 7.88 m.

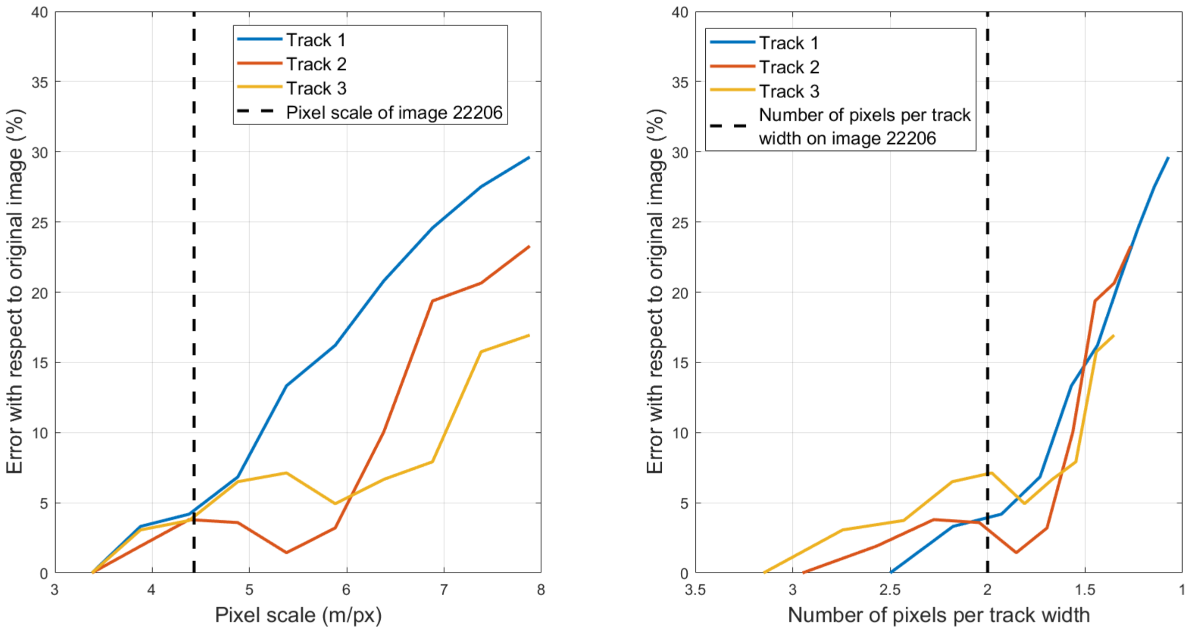

(a) (b)

Supplementary Figure 5. Error in the track width measurement between the original image of Didymos and the degraded image for each degraded resolution. The error is shown as a function of (a) pixel scale and (b) the number of pixels per track width. The vertical dashed line shows the resolution of the DRACO image of Didymos used for this study.

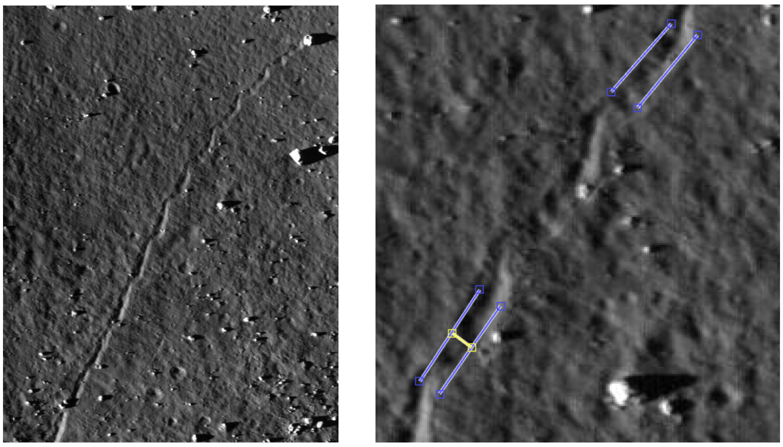

(a) (b)

Supplementary Figure 6. (a) Lunar track studied on image M135215829RC with a pixel scale of 0.5 m. (b) The edges are marked (in purple) and an example width measurement is also provided (in yellow).

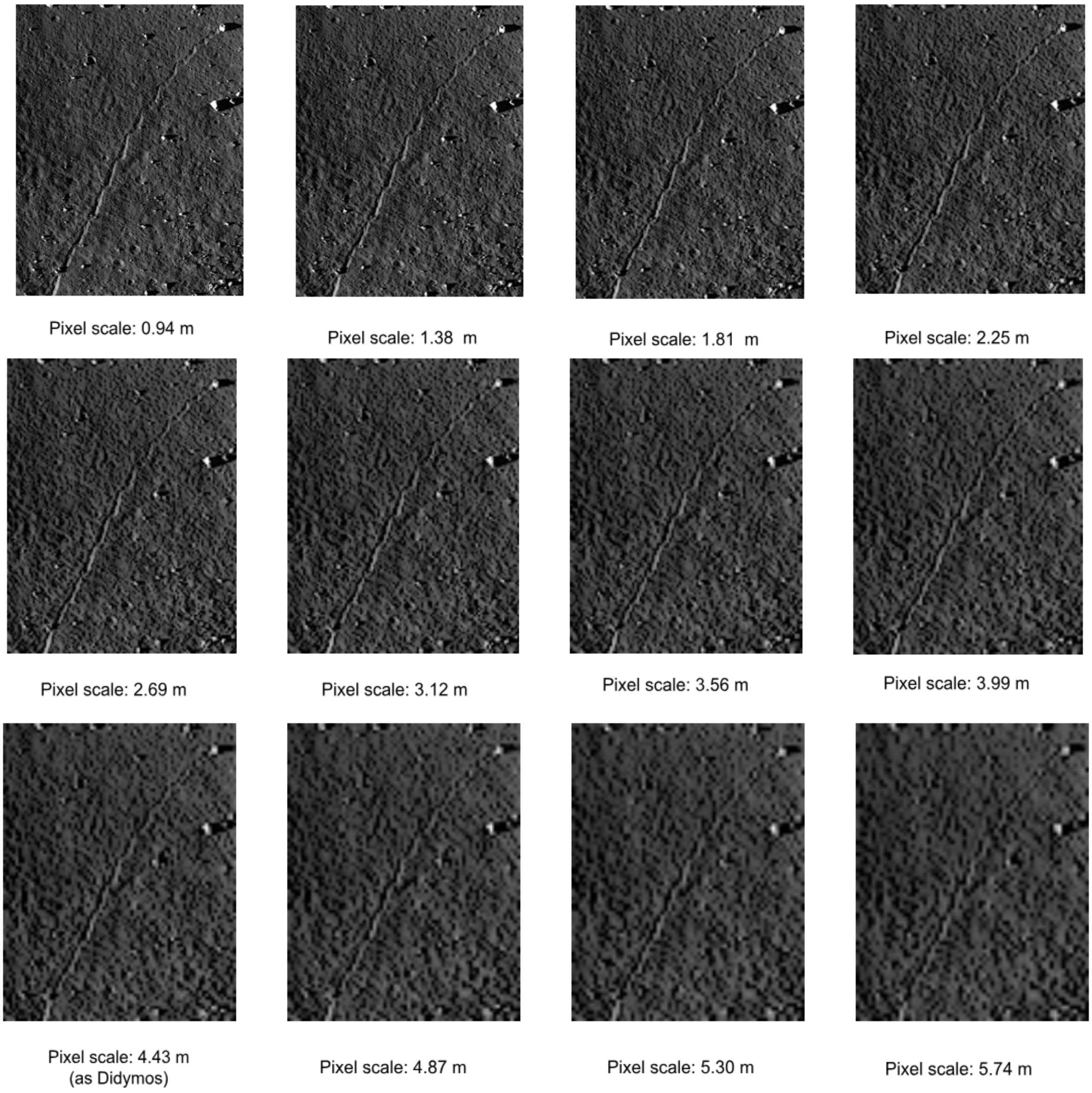

Supplementary Figure 7. Degraded versions of the lunar image (Supp. Fig. 6) with different pixel scales.

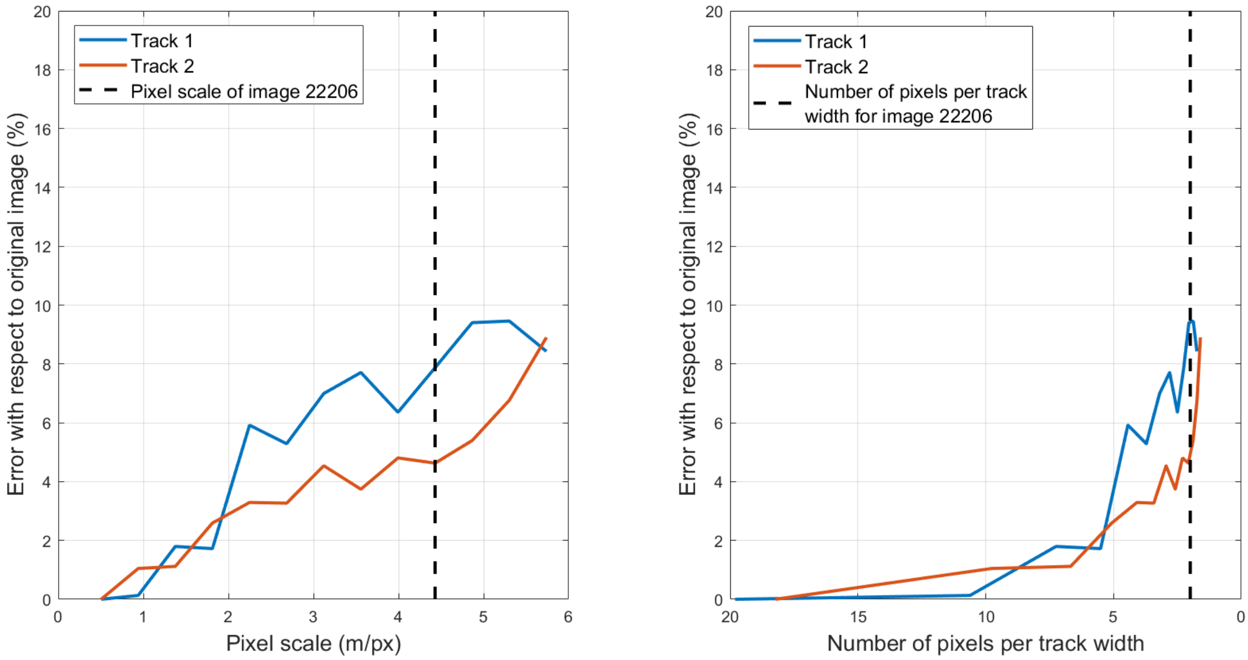

Supplementary Figure 8. Error in the track width measurement between the original lunar image and the degraded images. The error is shown as a function of the number of pixels per track width. The vertical dashed line shows the number of pixels per track width in the DRACO image of Didymos used for this study (image 22206).

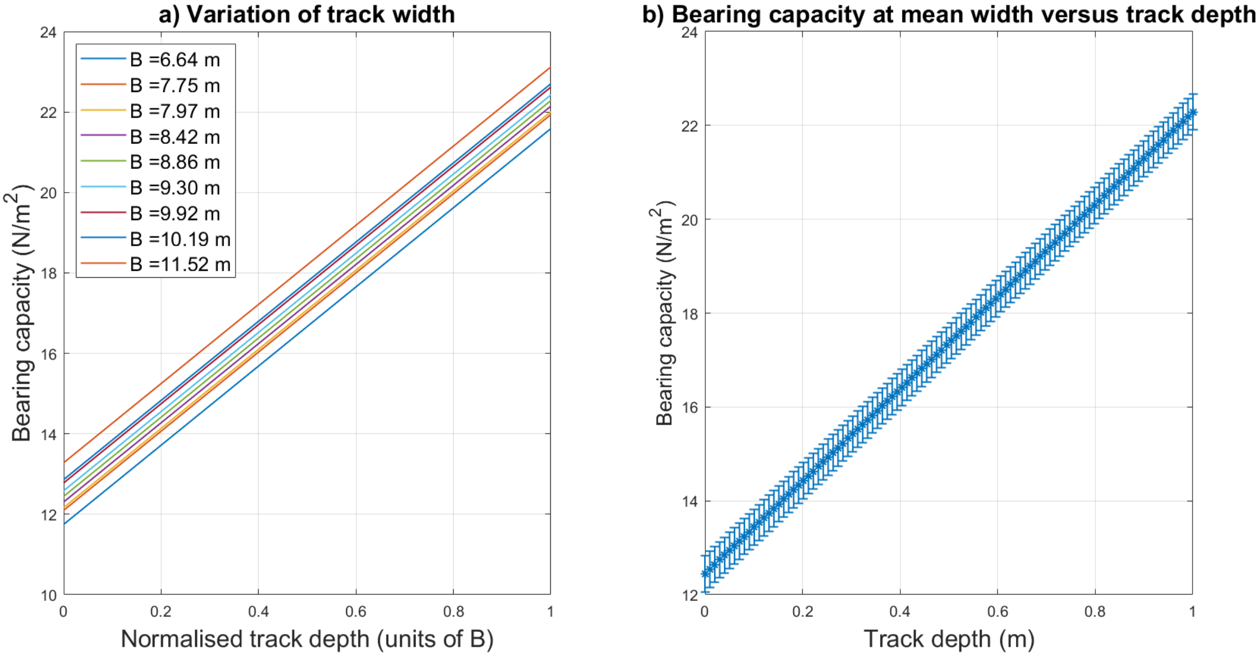

(a) (b)

Supplementary Figure 9. (a) Influence of varying the track width (measurements made on image 22206) while keeping the angle of internal friction, cohesion, gravity and regolith density fixed. (b) Mean bearing capacity as a function of the track depth D $\in$ [0; B]. The markers show the bearing capacity for the mean width ($\overline{B}$) and the error bars show the range of values for different track widths (i.e., $\overline{B} = 8.9 \ \pm 1.5\ m$). Here we assume an angle of internal friction of 35°, a cohesion of 1 N/m^2, and a local effective gravity of 3.1 x 10^{-5} m/s^2 at the mean latitude of 13° (GM = 35.4 m^3/s^2, R = 394 m, T = 2.26 hrs).

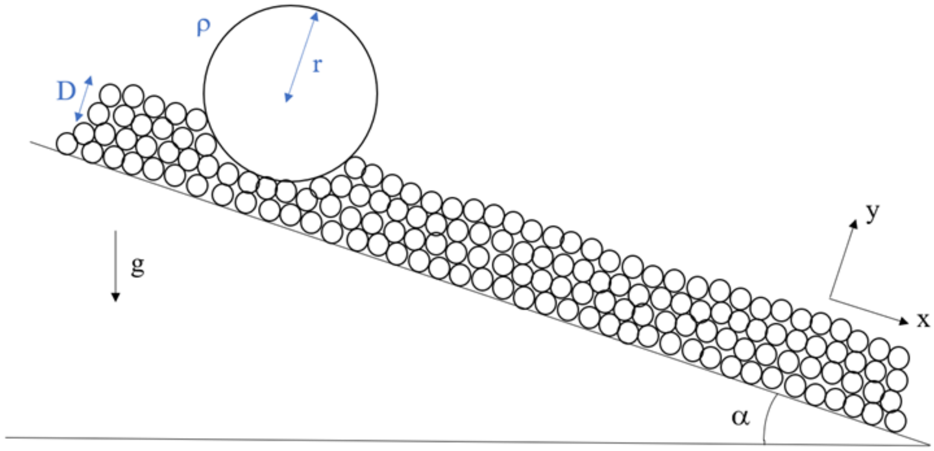

Supplementary Figure 10. Schematic of the simplified situation: a boulder of radius *r* and density ρ penetrates to a depth *D* while rolling (or sliding) along an inclined plane of slope α covered with a

granular material under the influence of the gravitational acceleration, g. After [38]Darbois Texier et al., (2018).

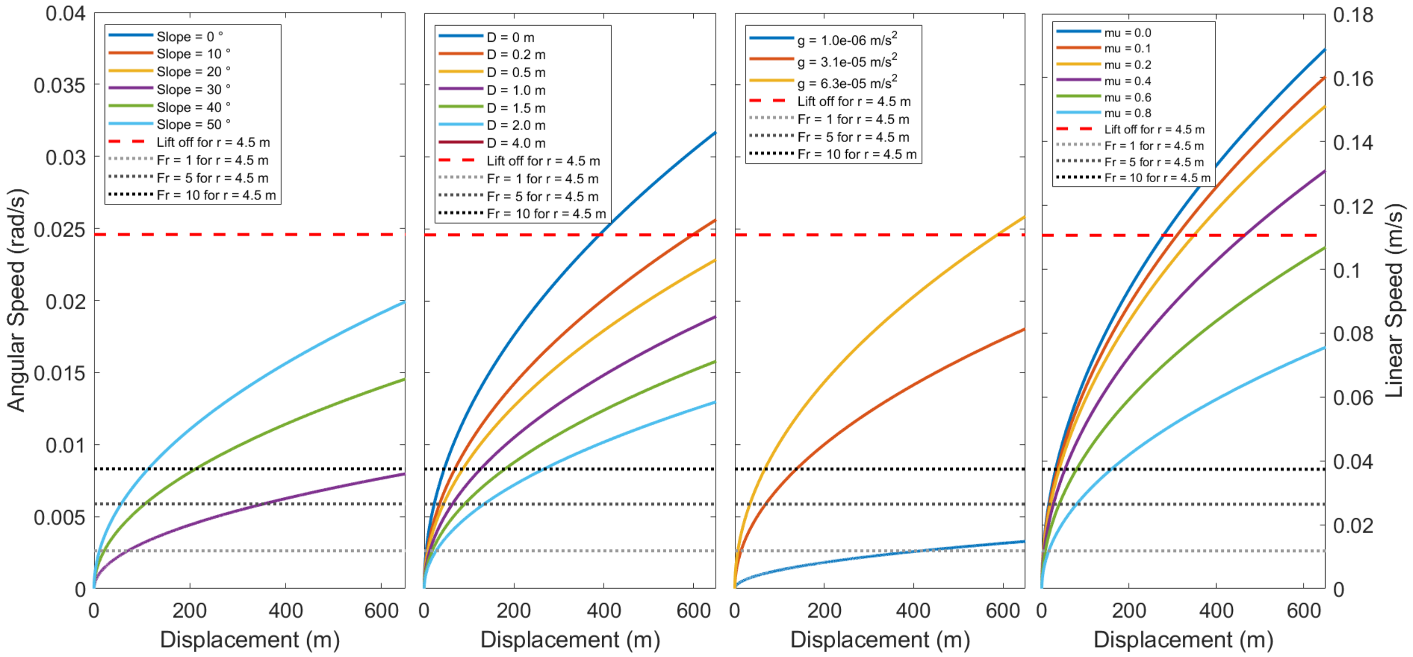

(a) **(b)** **(c)** **(d)**

Supplementary Figure 11. Speeds of a 4.5 m radius boulder on the surface of Didymos as a function of the distance travelled. In the case of a rolling boulder, the influence of slope α, track depth D, effective gravity g_{eff}, on the angular and linear speeds of the boulders is shown in (a), (b) and (c), respectively. In the case of a sliding boulder, the influence of the dynamic friction coefficient μ_d on the linear speed is shown in (d). The parameters are varied individually while keeping the others equal to the baseline values of α = 45°, D = r/4 = 1.125 m, g_{eff} = 3.1 x 10^{-5} m/s^2 (GM = 35.4 m^3/s^2, R = 394 m, T = 2.26 hrs, latitude = 13°) and boulder density ρ = 2790 kg/m^3**.** In (a) the slope is varied from 0° to 50° but there is no displacement below the critical slope (30° here). The horizontal red dashed line shows the theoretical speed at which a 4.5 m radius boulder would lift off the surface of Didymos. The horizontal light grey, dark grey and black lines show the angular Froude numbers of 1, 5 and 10 respectively for a 4.5 m radius boulder.

`

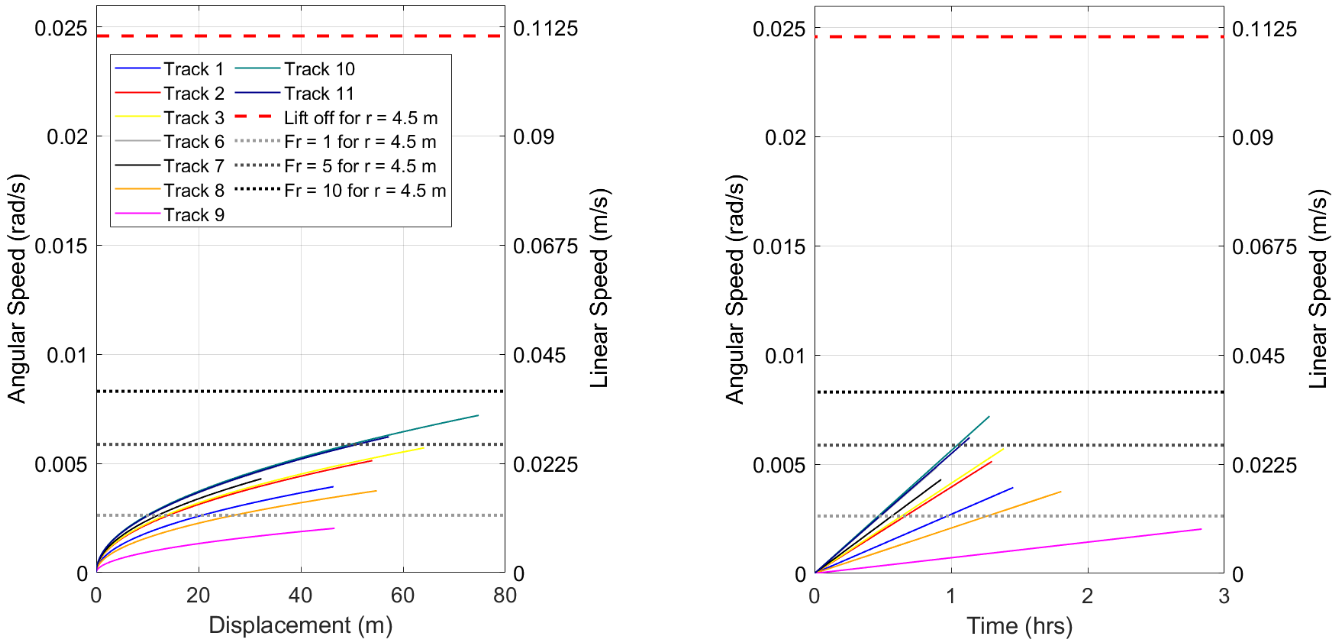

(a) (b)

Supplementary Figure 12. Angular speed of a 9 m diameter boulder along the boulder tracks on the surface of Didymos as a function of the (a) displacement and (b) time spent rolling. We consider for each track its mean slope, mean latitude and its length. The modification in the effective gravitational acceleration due to the asteroid's rotation and oblateness is taken into account. The following baseline parameters are assumed: D = 1.125 m, GM = 35.4 m^3/s^2, R = 394 m, T = 2.26 hrs. The horizontal dashed lines show the theoretical speed at which a 4.5 m radius boulder would lift off the surface of Didymos. The horizontal light grey, dark grey and black dotted lines show the angular Froude numbers of 1, 5 and 10 respectively for a 4.5 m radius boulder.

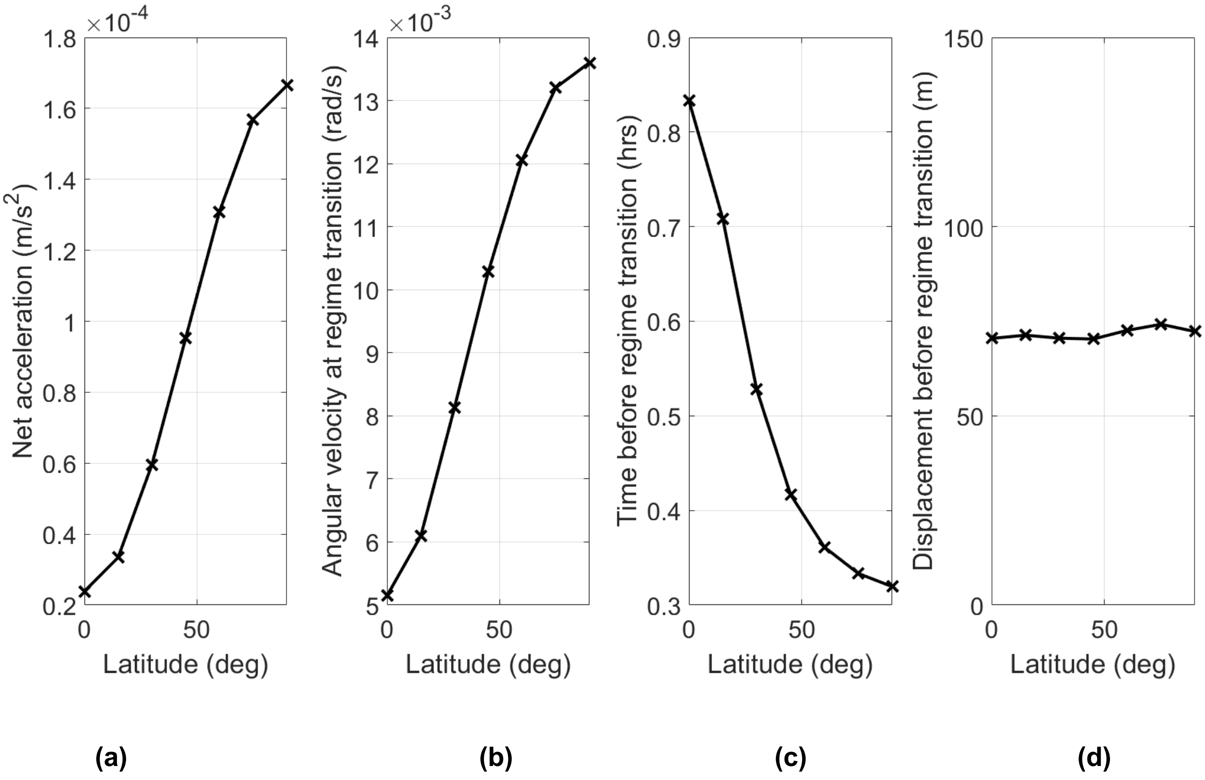

Supplementary Figure 13. Influence of the latitude on a rolling boulder. Influence of latitude on the (a) net surface acceleration (also referred to as the effective gravitational acceleration), (b) angular velocity of the regime transition, (c) time spent rolling before the regime transition, (d) displacement before the regime transition. The following baseline parameters are assumed: r = 4.5 m, α = 45°, D =1.125 m, GM = 35.4 m^3/s^2, R = 394 m, T = 2.26 hrs, and the regime transition occurs at Fr = 5.

# - Supplementary Tables -

Track number	Width in raw image 10933 (m)	Width in filtered image 10933 (m)	Difference between raw and filtered image (%)	Width in raw image 05363 (m)	Width in filtered image 05363 (m)	Difference between raw and filtered image (%)	Width in raw image 22206 (m)	Width in filtered image 22206 (m)	Difference between raw and filtered image (%)
1	7.0	7.2	2.9	8.5	8.5	0.0	8.2	8.4	2.4
2	8.4	8.3	1.2	10.65	10.0	6.5	9.7	9.9	2.1
3	9.3	9.2	1.1	10.7	10.6	0.9	9.9	10.2	3.0

Supplementary Table 1. Measurements (in m) of track widths in raw and filtered images using partial image 10933 (pixel scale: 3.47 m), partial image 05363 (pixel scale: 3.38 m) and full image 22206 (pixel scale: 4.43 m).

Track number	Width in filtered image 10933 (m)	Width in filtered image 05363 (m)	Width in filtered image 22206 (m)	Maximum difference (%)
1	7.2	8.5	8.4	18.1
2	8.3	10.0	9.9	20.5
3	9.2	10.6	10.2	15.2

Supplementary Table 2. Measurements (in m) of track widths in filtered images with different resolutions: image 10933 (pixel scale: 3.47 m), image 05363 (pixel scale: 3.38 m) and image 22206 (pixel scale: 4.43 m).

Resolution (m/px)	Track 1 width (m)	Difference with respect to original image (%)	Track 2 width (m)	Difference with respect to original image (%)	Track 3 width (m)	Difference with respect to original image (%)	Mean difference per resolution (%)
7.88	10.9	29.6	12.3	23.3	12.5	17.0	23.3
7.38	10.8	27.5	12.0	20.6	12.3	15.8	21.3
6.88	10.5	24.6	11.9	19.4	11.5	7.9	17.3
6.38	10.2	20.1	11.0	10.1	11.4	6.7	12.3
5.88	9.8	16.2	10.3	3.2	11.2	4.9	8.1
5.38	9.6	13.3	10.1	1.4	11.4	7.1	7.3
4.88	9.0	6.8	9.6	3.5	9.9	6.5	5.6
4.38	8.8	4.2	9.5	3.8	10.2	3.7	3.9
3.88	8.7	3.3	10.2	1.9	10.3	3.1	2.8
3.38	8.5	0	10.0	0	10.6	0	0

Supplementary Table 3. Measurements of track widths (in m) in image 05363 (pixel scale: 3.38 m) and difference between initial and degraded images.

Resolution (m/px)	Track 1 length (m)	Difference with respect to original image (%)	Track 2 length (m)	Difference with respect to original image (%)	Track 3 length (m)	Difference with respect to original image (%)	Mean difference per resolution (%)
7.88	51.06	15	39.2	13	41.2	22.1	16.7
7.38	52.8	19	40.4	16	40.7	20.4	18.5
6.88	47.2	6.1	37.9	9.0	42.6	26.1	13.8
6.38	57.7	30	42.4	22	41.2	22.0	24.7
5.88	47.2	6.2	36.6	5.3	34.3	1.5	4.3
5.38	48.5	8.9	40.0	15	33.7	0.28	8.1
4.88	46.4	4.3	34.7	0.24	38.1	13.0	5.8
4.38	43.4	4.4	39.6	14	37.9	12.3	10.2
3.88	48.9	10	43.3	24	37.7	11.7	15.2
3.38	44.5	0	34.8	0	33.8	0	0

Supplementary Table 4. Measurements of track lengths (in m) on image 05363 (pixel scale: 3.38 m) and difference between initial and degraded images.
